# A neo-taphonomic approach to human campsites modified by carnivores

**DOI:** 10.1038/s41598-020-63431-8

**Published:** 2020-04-20

**Authors:** Maite Arilla, Jordi Rosell, Ruth Blasco

**Affiliations:** 1 Àrea de Prehistòria, Universitat Rovira i Virgili (URV), Avinguda de Catalunya, 35, 43002 Tarragona, Spain; 2 IPHES; Institut Català de Paleoecologia Humana i Evolució Social, Zona Educacional 4, Campus Sescelades URV (Edifici W3), 43007 Tarragona, Spain; 3 Centro Nacional de Investigación Sobre La Evolución Humana (CENIEH), Paseo Sierra de Atapuerca 3, 09002 Burgos, Spain

**Keywords:** Behavioural ecology, Palaeoecology

## Abstract

Skeletal profiles at archaeological bone assemblages can bear little resemblance to original hominin discarded bone elements. Resulting patterns might originate from different taphonomic problems, such as hominin-carnivore activities in alternate visits, and lead to interpretation issues. In this paper, we present a study of predepositional scattering activities caused by small-sized carnivores on simulated short-term hominin campsites. Their disrupting actions affect skeletal element survival considerably and, to a lesser extent, the spatial distribution of hearth-related assemblages. The results of this study demonstrate that small-sized carnivores might cause as much disruption as large-sized ones. Thus, being able to recognize these taphonomic processes and their consequences is critical when discerning between human and non-human behaviour.

## Introduction

The intrusion of carnivores as secondary consumers at abandoned human camps is a common phenomenon that has been noted in several actualistic works (e.g.,^1–8^). All observations have suggested that potential perturbations caused by these animals can overprint the composition and original spatial location of artefacts and faunal remains that make up an assemblage. Unfortunately, many archaeological studies are still conducted without considering these possible incidences in the final features of preserved assemblages, causing subsequent misinterpretations.

Binford^1,2^, one of the first researchers who described this phenomenon, noted regular visits of wolves to abandoned campsites, which may have been attracted by the mixture of smells generated by the combination of hearths and refuse of consumed carcasses, both cooked and raw. The lack of humans in the vicinity represented, for these scavengers, an opportunity to obtain food in a fast and easy way. According to Binford^1,2^, these intruders looked for specific and quickly ingestible elements (e.g., long bone epiphyses) and the regular transport of larger items beyond the boundaries of the campsite due to fear of a possible sudden return of humans. His results indicated a significant bias in the original assemblages with the disappearance of many items and frequently altered spatial positioning of many others. Similar behaviour was observed by Isaac^4^ among hyenas and jackals during fieldwork in Olorgesailie, Kenya. This experience helped Isaac raise several aspects of those archaeological sets, describing this phenomenon by deliberately dumping bones from domestic refuse in specific areas outside the campsite. At the end of the season, many bones had been removed by the scavengers, and only some mid-shaft fragments with *a priori* lower nutritional value were recovered. For these authors, the implications of these phenomena must always be considered during archaeological interpretation, and they concluded that only accumulations where bones and lithic artefacts followed similar spatial patterns could be considered undisturbed by scavengers^5^.

Following the idea further, Binford *et al*.^9^ developed two experimental series in an African context. They began by dumping a combination of broken and intact defleshed buffalo limb bones in two specific places: one close to their own campsite and one in a more remote area. Both assemblages attracted a succession of scavengers at different times, such as marabou storks, ground hornbills, jackals and hyenas. All these scavengers had a significant preference for marrow over other edible elements, such as meat. For example, the researchers noted a regular disappearance of the broken bones still containing marrow, which were displaced by several tens of metres, and a high percentage of fractures on previously unbroken limb bones. Conversely, broken bones without marrow (mainly mid-shaft fragments and splinters) remained practically intact and close to their original locations, even those with intact soft tissue attached to their ends. Another aspect observed by these researchers was the swiftness with which the scavengers acted. Although the assemblages were monitored for several days, the most significant damage was generated during the first 24 hours. Regarding spatial patterns, Binford *et al*.^9^ highlighted remarkable perturbations. Most of the carnivore-damaged bones were scattered in peripheral areas far from the original areas where they were dumped. By contrast, only small fragments remained in the original zones without carnivore damage.

Trying to decipher which agent (hominins or carnivores) acted first in the African Early Pleistocene sites, Blumenschine^10^ exposed several assemblages composed of whole and broken limb bones to wild carnivores, mainly spotted hyenas and lions of the Serengeti. Again, these experiments revealed carnivore interest in bone ends and a low number of tooth-marked elements, whose rates did not reach 15% (or even less) in riparian habitats. Marean and Spencer^11^ used the data provided by Blumenschine^10^ in comparison with captive spotted hyenas. These authors exposed 33 defleshed broken and unbroken bones to the action of these animals in a simulated scenario of abandoned human settlement. A high degree of damage was observed on axial bones; ribs and vertebrae rarely survived and only 50% of pelvises were recovered. Regarding limb bones, all observations fully coincided with previous works showing a high proportion of damage observed on epiphyses. By contrast, all femur and tibia mid-shafts remained untouched, and tooth marks were only recorded on some metatarsals. This line of research was recently resumed by Arriaza *et al*.^12^ in a still incipient work, demonstrating how the intervention of many variables could condition carnivore actions in this kind of scenario. They exposed a previously processed domestic goat to scavengers during three nights in an area near the FLK site in Olduvai (Tanzania). The carcass was only disturbed by a single striped hyena that was feeding constantly in the same place for two nights. No transporting of bones outside the area was recorded. However, the results were similar to previous observations: disappearance of limb bone epiphyses and axial bones, a lack of interest in broken diaphyses, little damage on recovered bones and significant movements and reorientation of bone fragments, intentional and unintentional (by trampling).

Faith *et al*.^13^ conducted a series of 33 experimental reproductions of carnivore-modified archaeofaunal assemblages at the Berkeley Spotted Hyena Colony. In general, their results did not differ from previous works: significant disappearance of epiphyses and little interest in diaphysis splinters. However, a main contribution of this work was the relationship established between bone density and the carnivore behaviour. A clear correlation between bone density and long-bone portion survivorship was observed in absence of competition (that is, one hyena or several individuals with a similar hierarchical range). However, Faith *et al*.’s^13^ results varied significantly when an involved carnivore displayed a dominant position. In such cases, dominant individuals tended to eat at the place, while non-dominant individuals tried to transport bones indiscriminately away without considering the bone portion (dense or otherwise). For transported portions, density testing did not yield significant results.

All of the previously cited works focused primarily on the interpretation of archaeological assemblages without fire. However, as Binford^1,2^ observed, carnivore ravaging activities are also common in current and subcurrent contexts where fire played a central role. This technology introduced a new behaviour into human groups and new perceptions of space early in our evolutionary history^1,14–16^. At the ethnographic level, several researchers provided descriptions of carnivores as secondary consumers in the campsites of the Hadza and the!Kung San tribal groups^6,7,17,18^. These observations do not differ significantly from previous studies as carnivores preferred limb bone ends (which usually disappeared via ingestion or a high degree of fracturing), and the usual preservation of mid-shaft assemblages previously broken during human consumption processes because of a lack of attractors, such as marrow^10,17,19–21^. Following this line of research, Camarós *et al*.^22^ developed an experimental study with different kinds of carnivores (bears, hyenas, lions and wolves). The study reproduced several hearth-related areas in which various domestic activities were carried out (knapping, defleshing and roasting meat) and exposed them to these animals. The results indicated differential degrees of disturbance. Bears were the most active and moved practically all items, including stones, lithics and bones, while other carnivores modified the simulated campsites to lesser degrees. However, these observations were conducted in a park with semi-captive animals, making the results only informative of the potential capabilities of animals to modify anthropogenic assemblages. Here, we present a similar experiment with animals in a wild state. The primary objective is to produce data to model the carnivore behaviour of carnivores in these contexts and contribute to understanding the spatial dispersion and anatomical biases in archaeological assemblages with well-defined hearth-related areas.

## Methods

All experimental protocols were approved by the *Generalitat de Catalunya* (Catalan Government, Territory and Sustainability Department), through authorizations 118/2015, 12/2017, 11/2018, to develop scientific research and camping activities. All methods were carried out in accordance with current legislation and the following decrees: 1) Decree 194/2003, August 1st, of the creation of the Parc Natural de l’Alt Pirineu (DOGC [Official Journal of the *Generalitat de Catalunya*] N. 3943 of 08.08.2003) - Art. 3.8; 2) Law 12/1985, June 13th, of natural spaces (DOGC N. 556 of 06.28.1985) - Art. 37 and others; 3) Decree 148/92, June 9th, regulating photographic, scientific and sport activities that may affect wildlife (DOGC N. 1618 of 07.13.1992) - Art. 4.1 and 5.1.

The experimental series were conducted for three years, during which time a total of 10 campsites were set up in two different areas. Three experimental series (one each year) were carried out in the Pyrenees (Sector 1[S1]). This mountain range extends E-W for about 425 km from the Mediterranean Sea to the Cantabrian Sea, making a natural border between the Iberian Peninsula and France. The Alpine orogeny led to the formation of different geological features such as rock peaks (up to 3,000 metres high) or deep valleys characterised by a highline climate that affects flora in a gradient way depending on the height. The faunal diversity includes herbivores such as chamois (*Rupicapra rupicapra*), mouflon (*Ovis musimon*), roe deer (*Capreolus capreolus*), red deer (*Cervus elaphus*), fallow deer (*Dama dama*) and omnivores such as wild boar (*Sus scrofa*). Carnivores are represented by bears (*Ursus arctos*) and a great range of small species, with red fox (*Vulpes vulpes*), pine marten (*Martes martes*), and badger (*Meles meles*) being the most common. Today, the bear population in the Pyrenees is composed of 43 individuals. The presence of this species is the primary reason why this territory was chosen with the aim to characterise the possible impact of this large carnivore on the campsites.

**Figure 1:**
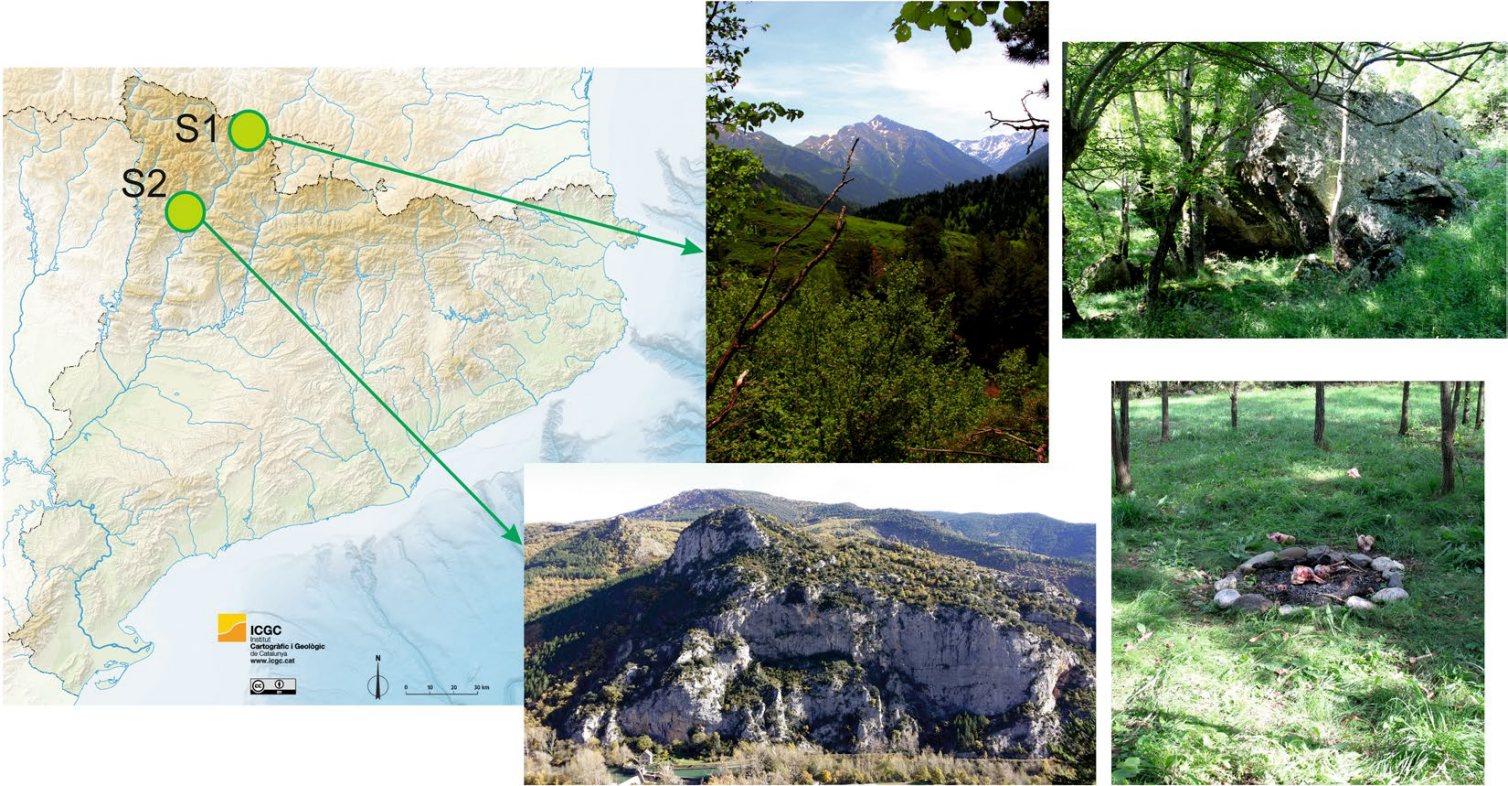
The Pyrenean Mountains in the context of the Iberian Peninsula depicting the location of the neo-taphonomic studies. Sector 1 (S1) is located in the Parc Natural de l’Alt Pirineu (North-Pyrenees, Pallars Sobirà, Spain), specifically in a rock-shelter between the Raspamala forest and the Clavera stream. Sector 2 (S2) is located in the Pallars Jussà region (South-Pyrenees, Spain). The map was obtained from an open access source of the Institut Cartogràfic i Geològic de Catalunya (ICGC) [https://www.icgc.cat/L-ICGC/Sobre-l-ICGC/Recursos-didactics/Mapes-fisics], used under a CC BY 4.0 license and prepared with the Adobe Photoshop CS5 Version 12.0.4 × 64 software.

The neo-taphonomic study was performed in the Parc Natural de l’Alt Pirineu (Pallars Sobirà, Spain), specifically in a rock-shelter between the Raspamala forest and the Clavera stream (Fig. 1). During six months of the year (December to June) the amount of accumulated snow makes it impracticable to access the primary path that crosses the area; therefore, the human presence at that time is non-existent. The work was conducted at the end of this semester (May to June), in which all the paths along the park are closed according to the park policy, in addition to the fact that these kinds of studies are only allowed during this time of the year due to a lack of tourism in the zone. The experimental work in Sector 1 was supervised by the support team (Bear Patrol) of the *Fundación Oso Pardo* (FOP, by its initials in Spanish).

The rest of experimental series (n = 7) was performed in Sector 2 (S2) in different seasons throughout three years. The study was conducted in a partially mountainous territory in the region of Pallars Jussà (South-Pyrenees, Spain), specifically in a woodland area crossed from North to South by the Flamisell River (Fig. 1). The mixed forest diversity includes trees such as pines (*Pinus* sp.), oaks (*Quercus* sp.), maples (*Acer* sp.), false acacia (*Robinia pseudoacacia*), black poplar (*Populus nigra*), willow (*Salix*), and ash tree (*Fraxinus*), but also patches of low vegetation consisting of a wide variety of riverside bushes like hawthorn (*Crataegus monogina*), blackthorn (*Prunus spinosa*), and box (*Buxus sempervirens*) combined with fields. Human population in the area is sparse; even so, the faunal diversity is significantly different compared to S1: herbivores are represented by roe deer (*Capreolus capreolus*) and red deer (*Cervus elaphus*), omnivores by wild boar (*Sus scrofa*), and small carnivores include the red fox (*Vulpes vulpes*), stone marten (*Martes foina*), genet (*Genetta genetta*) and badger (*Meles meles*). The rare human presence, together with the abundant presence of small-sized carnivores, was the reason why this study area was selected.

All the experimental series consisted of the replication of a campsite area (~30 m²) composed of a central hearth and the remains of food and lithic stone tools simulating a short-term human occupation. The main goal was to observe any modification inflicted by carnivores on the abandoned items and/or structures. Food remains included small and large-sized animals [class 2 and 4 *sensu*^20^]. Size class 2 (or small size) includes animals weighting between ~20 and 120 kg (e.g., sheep, lamb), and size class 4 (or large size) include ungulates with ~300–1000 kg (e.g., cattle). A quarter of a lamb (*Ovis aries*) was roasted and eaten each series and their bones (previously classified) distributed around the hearth once eaten. All individuals were about 4 months old and weighed approximately 20–25 kg. The remains of this species included both axial and appendicular skeletal elements. Simultaneously, different de-fleshed cow limb bones (*Bos taurus*), were fresh fractured for marrow extraction and deposited on the hearth-related area: some directly on the hearth and other ones on the closest areas to the hearth. In addition to this, one humerus and one hemi-pelvis were deposited whole. The bones came from 17 months old calves weighing about 450 kg. This skeletal profile is based on the main standards identified in some Middle Palaeolithic sites, in which a succession of short-term human occupations has been interpreted (e.g.,^23^).

All the bone fragments were numbered to verify possible displacements. Six stone tools were also numbered and placed around the combustive structure after using them in the dismembering/defleshing processes. Small wooden logs of the hearth were also mapped. The campsite was abandoned once the entire process had been documented and exposed to the carnivores.

All the elements and related-structures were recorded by means of a Trimble S6 total station (GPS Trimble R6 and GPS TwoNavSportiva receptors) and using VRS (Virtual References Station) technique. Apart from the official reference system, three reference points were settled to correlate measurements. The mapping was conducted using the MiraMon system (SIG). All series were registered with wildlife cameras so as to document the predators involved in any related activity inside and around the campsite. The photo/video-trap was carried out using No Glow Infrared Trail cameras that are equipped with invisible black LED (Black Flash). This technology does not produce the visible red glow as a side effect. Three wildlife cameras were placed around the campsites (S1 and S2) during the actualistic experiments. Weekly site reviews allowed us to report the perturbation/modification level of the site. Additionally, six cameras belonging to the support team of the *Fundación Oso Pardo* were also permanently placed in different locations of Sector 1 on a range from 300 metres to 3.1 km. to determine the presence/absence of the brown bear nearby the campsite. Eventually, the removal of the remains was carried out when prolonged lack of visits by carnivores indicated the end of predators’ interest using the same initial recording process. In addition to this, an area of about 20,000 square metres was searched in detail to record possible long-distance displacements.

All skeletal remains were cleaned if necessary using non-aggressive techniques such as boiling in a solution of water and non-enzymatic detergent. Resistant tissues were removed by using a wooden knife or soft pointed tools that did not compromise the bone surface. Bone damage on the recovered specimens was analyzed using an Olympus SZ 11 stereoscopic (magnification up to 110×). The categorisation of tooth marks such as pits/punctures and scores has been effectuated following the classification of Haynes^24–26^, Binford^2^, Blumenschine^27^, and Maguire *et al*.^28^. Different bone modifications like furrowing^2,24,26,28^, crushing^2^ or fractures^2,28^ have also been identified and recorded. A range of measures for pits and punctures has been established^29–32^.

A contingency table (χ^2^) and a correspondence analysis (CA) have been calculated to determine the dependence degree of the analyzed variables and the diversity of the observations (OBs). In addition, the relationship between presence/absence of bone portions and their density has been checked using a parametric correlation coefficient (Pearson *r*). The cattle and lamb bones have been treated separately: cattle specimens were calculated with bone-density data provided by Ioannidou^33^, and lamb specimens with the data from Lyman^34^, which provided values of axial remains.

## Results

Ten simulated campsite areas were set up over three years in two different areas of the Spanish Pyrenees (Sector 1[S1] and Sector 2 [S2]; Fig. 1). A total of 60 stone tools were placed together with 466 skeletal elements. The bone assemblage consisted of large-sized (n = 131) and small-sized skeletal elements (n = 335) belonging to a MNI of 20 (MNI = 10 of size class 4 and MNI = 10 of size class 2). From the total sum, 388 (83.26%) elements disappeared and a set of 78 (16.74%) anatomical elements were recovered. The recovered MNI (n = 14) indicates a bias towards size class 4 (n = 10) in contrast to the recovered MNI of size class 2 (n = 4). The largest part of the recovered items belongs to large-sized animals (n = 57; 43.51% of placed bones from the same size class), specifically mid-shaft fragments (n = 51; 38.93%). Only 6.27% (n = 21) of recovered bones belong to size class 2 (Table 1). Of these recovered bones, 14 (17.95%) exhibited modifications produced by small carnivores, with percentages of 10.26% (n = 8) of size class 2 and 7.69% (n = 6) of size class 4, mostly located on mid-shaft fragments of long bones (n = 5; 6.41% related to recovered), followed by scapula (n = 3; 3.85%) and ulna (n = 2; 2.56%). The rest of modifications were located on proximal epiphysis of long bones (size class 4), humerus, radius, vertebrae and ribs (size class 2) with the same rates (n = 1; 1.28%). Regarding anatomical regions, appendicular elements present an 11.54% (n = 9) of bone modifications followed by girdles (3.85%; n = 3) and axial skeleton (2.56%; n = 2). S2 displays higher carnivore activity with 38 (11.11%) recovered bones and 18.42% (n = 7) of bone damage while 40 skeletal elements (32.26%) were recovered at S1, demonstrating a 17.50% (n = 7) of bone-inflicted damage. The percentage of missing bones in S2 reaches 88.89% (n = 304) in contrast to a 67.74% (n = 84) in S1 (Table 2).

**Table 1:** General characteristics of the observation periods (OBs).

			OB 1	OB 2	OB 3	OB 1	OB 2	OB 3	OB 4	OB 5	OB 6	OB 7	Total
**Sector [S]**			S1	S1	S1	S2	S2	S2	S2	S2	S2	S2	
**Season + year**			Spring '16	Summer '17	Spring '18	Spring '16	Autumn '16	Winter '17	Summer '17	Winter '18	Summer '18	Summer '18	
**Main carnivore**			*V. vulpes*	*V. vulpes*	*V. vulpes*	*V. vulpes*	*V. vulpes*	*V. vulpes*	*V. vulpes*	*V. vulpes*	*V. vulpes*	*V. vulpes*	
**Secondary predator/scavenger**			*M. martes*	*U. arctos M. martes*	*M. martes*				*M. foina*	*M. foina G. genetta B. buteo G. fulvus*	*M. foina*	*M. foina S. scrofa*	
**Placed bones**	*Bos taurus*	Prox. epiph [HUM, FEM, TB]	3	2	2	3	3	3	3	2	3	3	27[HUM(3),FEM(15),TB(9)]
		Dist. epiph [HUM, FEM, TB]	3	2	2	3	3	3	3	2	3	3	27[HUM(3),FEM(15),TB(9)]
		Mid-shafts [HUM, FEM, TB]	12	7	4	9	8	8	9	3	7	8	75[HUM(7),FEM(37),TB(31)]
		Hemi-pelvis		1									1
		Humerus								1			1
	*Ovis aries*	Scapula	1	2		2	2	1	1	1	1	1	12
		Humerus	1	2	1	2	2	1	1	1	1	1	13
		Radius	1	2	1	2	2	1	1	1	1	1	13
		Ulna	1	2	1	2	2	1	1	1	1	1	13
		Ribs	12	12	11	11	15	13	12	20	6	26	138
		Vertebrae	8	11	17	8	14	16	14	21	9	27	145
		Sternum				1							1
**Total**			42	43	39	43	51	47	45	53	32	71	466
**Recovered bones**	*Bos taurus*	Prox. epiph [FEM, TB]		2					1				3[FEM(2),TB(1)]
		Dist. epiph [FEM, TB]		2									2[FEM(1),TB(1)]
		Mid-shafts [HUM, FEM, TB]	11	6	1	5	8	2	9	2	3	4	51[HUM(3),FEM(24),TB(24)]
		Hemi-pelvis		1									1
	*Ovis aries*	Scapula		2				1	1				4
		Humerus		2									2
		Radius		2		1							3
		Ulna		2		1							3
		Ribs		6									6
		Vertebrae		3									3
**Total**			11	28	1	7	8	3	11	2	3	4	78
**Damaged bones**	*Bos taurus*	Prox. epiph [FEM]							1				1[FEM(1)]
		Mid-shafts [FEM, TB]	1		1		1			1	1		5[FEM(3),TB(2)]
	*Ovis aries*	Scapula		1				1	1				3
		Humerus		1									1
		Ulna		1		1							2
		Ribs		1									1
		Vertebrae		1									1
**Total**			1	5	1	1	1	1	2	1	1		14

HUM = Humerus; FEM = Femur; TB = Tibia; epiph = epiphysis.

**Table 2:** Carnivore activities split by size-class and sectors.

		Placed bones (*n*)	Recovered bones (*n*)	%	Absences (*n*)	%	Damaged bones (*n*)	%
**Sector 1**	*Bos taurus*	38	23	60.53	15	39.47	2	5
	*Ovis aries*	86	17	19.77	69	80.23	5	12.5
Total		124	40	32.26	84	67.74	7	17.5
**Sector 2**	*Bos taurus*	93	34	36.56	59	63.44	4	10.53
	*Ovis aries*	249	4	1.61	245	98.39	3	7.89
Total		342	38	11.11	304	88.89	7	18.42

As mentioned above, the lamb bones were systematically roasted, de-fleshed, and distributed without breaking around the hearth. On the contrary, almost all bones belonging to *Bos taurus* (n = 131) were broken and cleaned of marrow except for one humerus and one hemi-pelvis that were deposited whole. Among these skeletal elements, 45 were cooked and 86 were deposited raw (Table 3). The recovered bones did not show a preference regarding their thermo-alteration state, with 19 and 38 recovered items for both cooked and raw skeletal elements respectively (42.22% and 44.19% according to placed bones by size class and condition). However, 21.05% of alterations (n = 4) is registered on cooked bones in contrast with 5.26% (n = 2) on raw bones. Cooked bones correspond to mid-shaft fragments of long bones, while one proximal epiphysis and a mid-shaft fragment of a femur are the total of damaged raw bones.

**Table 3:** *Bos taurus* bones and their thermal condition (cooked/raw) during the experimental series.

	Skeletal element/region	Sector 1		Sector 2		Total
		Cooked bones	Raw bones	Cooked bones	Raw bones	
**Placed bones**	**Prox. epiph**	2[FEM]	5[FEM(3),TB(2)]	7[FEM(4),TB(3)]	13[FEM(6),TB(4),HUM(3)]	27 [FEM(15),TB(9),HUM(3)]
	**Dist. epiph**	3[FEM]	4[FEM(2),TB(2)]	7[FEM(4),TB(3)]	13[FEM(6),TB(4),HUM(3)]	27 [FEM(15),TB(9),HUM(3)]
	**Mid-shafts**	6[FEM]	17[FEM(8),TB(9)]	18[FEM(9),TB(9)]	34[FEM(14),TB(13),HUM(7)]	75 [FEM(37),TB(31),HUM(7)]
	**Hemi-pelvis**	1				1
	**Humerus**			1		1
**Total**		12	26	33	60	131
**Recovered bones**	**Prox. epiph**		2[FEM(1),TB(1)]		1[FEM]	3 [FEM(2),TB(1)]
	**Dist. epiph**	1[FEM]	1[TB]			2 [FEM(1),TB(1)]
	**Mid-shafts**	4[FEM]	14[FEM(5),TB(9)]	13[FEM(8),TB(5)]	20[FEM(7),TB(10),HUM(3)]	51 [FEM(24),TB(24),HUM(3)]
	**Hemi-pelvis**	1				1
**Total**		6	17	13	21	57
**Damaged bones**	**Prox. epiph**				1[FEM]	1 [FEM(1)]
	**Mid-shafts**	1[FEM]	1[FEM]	3[FEM(1),TB(2)]		5 [FEM(3),TB(2)]
**Total**		1	1	3	1	6

HUM = Humerus; FEM = Femur; TB = Tibia; epiph = epiphysis.

When split by season, both looting actions and bone damage were typically recorded in winter, with 95% (n = 95 of placed bones *per* season) of missing bones and 40% (n = 2 related to recovered bones *per* season) of bone alteration inflicted by small carnivores during this season, followed by spring with 84.68% (n = 105) and 15.79% (n = 3), and autumn with 84.31% (n = 43) and 12.5% (n = 1) respectively. The scattering activity declines significantly in the summer season (n = 145; 75.92%) while bone inflicted damage reaches 17.39% (n = 8) (Table 4). Regarding size class categories, the higher percentages of missing bones belong to small-sized animals in autumn (100%), winter (98.72%) and spring (97.59%), followed by summer (86.86%). On the other hand, large-sized animal bones are highly dispersed in winter season (81.82%), followed by spring (58.54%) and to a significantly lesser degree in summer (48.15%) and autumn (42.86%). Bone damage reaches 20% (n = 1) in both size categories in winter. Large sized bones show 12.5% (n = 1) bone inflicted damage in autumn, followed by 10.53% (n = 2) in spring and a percentage of 4.35% (n = 2) in summer, while bones of small size show higher modification in summer with 13.04% (n = 6) followed by spring (5.26%; n = 1).

**Table 4:** Carnivore’s activities split by size-class and season.

		Placed bones (*n*)	%	Recovered bones (*n*)	%	Absences (*n*)	%	Damaged bones (*n*)	%
**Spring**	*Bos taurus*	41	33.06	17	41.46	24	58.54	2	10.53
	*Ovis aries*	83	66.94	2	2.41	81	97.59	1	5.26
Total		124	26.61	19	15.32	105	84.68	3	15.79
**Summer**	*Bos taurus*	54	28.27	28	51.85	26	48.15	2	4.35
	*Ovis aries*	137	71.73	18	13.14	119	86.86	6	13.04
Total		191	40.99	46	24.08	145	75.92	8	17.39
**Autumn**	*Bos taurus*	14	27.45	8	57.14	6	42.86	1	12.5
	*Ovis aries*	37	72.55			37	100		
Total		51	10.94	8	15.69	43	84.31	1	12.5
**Winter**	*Bos taurus*	22	22	4	18.18	18	81.82	1	20
	*Ovis aries*	78	78	1	1.28	77	98.72	1	20
Total		100	21.46	5	5	95	95	2	40

**Figure 2:**
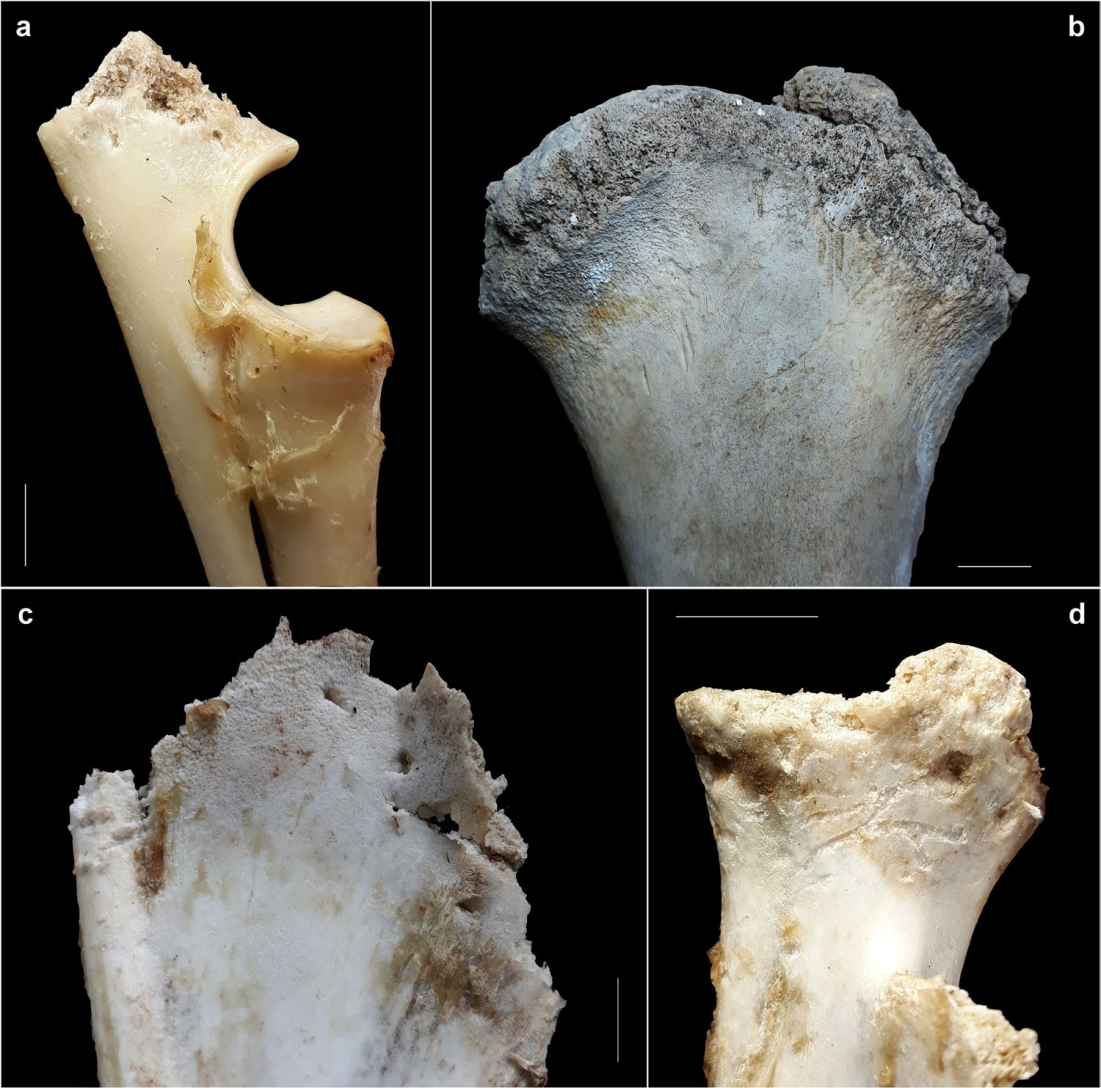
Examples of small carnivores-induced bone damage: (**a**) furrowing and pits on the olecraneum of an ulna (S2/BO1); (**b**) furrowing on the proximal epiphyses of a femur (size class 4)(S2/OB4); (**c**) co-occurrence of tooth-marks and crenulated edges on a scapula (S2/OB3); (**d**) pits, punctures and scores on the neck of a scapula (S2/OB3). Scale= 1 cm.

Bone damage in the form of pits, crenulated edges, furrowing, crushing, and longitudinal cracks have been identified (Table 5, Fig. 2). The most significant damages are pits and scores, which have been recorded in 12 items (15.38% of recovered bones). Pits are registered on 9 bones (11.54%), scores on 6 bones (7.69%), while 3 bones (3.85%) exhibited both alterations. Tooth marks affect both size class animals with percentages of 8.97 (n = 7) in relation to recovered bones and 6.41% (n = 5) on small and large-sized skeletal elements respectively, mostly located on the scapula (n = 2) and ulna (n = 2) for size class 2 and on the mid-shaft (n = 5) of long bone elements belonging to size class 4. Most of the tooth mark sizes are contained within a range of less than 3 × 2.5 mm, with averages of 1.75 mm on thick cortical, 1.79 on thin cortical, and 1.55 on cancellous tissue. Scores were located only on thick and thin cortical tissue with breadth averages of 0.85 mm and 0.6 mm respectively.

**Table 5:** Recovered MNI and damage identified on bone remains (NISP) split by sector (S) and observation period (OB). ^∗^Recovered..

Sector/OB	MNI*	NISP	Skeletal element	Pits *n*(%)	Scores *n*(%)	Furrowing *n*(%)	Crenul. edge *n*(%)	Crushing *n*(%)	Long. cracks *n*(%)
S1/OB1	1	11	Femur	1 (9.09)					
S1/OB2	2	28	Scapula	1 (3.57)	—	—	—	—	—
			Humerus	—	1 (3.57)	—	—	—	—
			Ulna	—	1 (3.57)	—	—	—	—
			Vertebra	1 (3.57)	—	—	—	—	—
			Rib	1 (3.57)	—	—	—	1 (3.57)	—
S1/OB3	1	1	Femur	1 (100)	—	—	—	—	—
S2/OB1	2	7	Ulna	1 (14.29)	—	1 (14.29)	—	—	—
S2/OB2	1	8	Tibia	1 (12.5)	1 (12.5)	—	—	—	—
S2/OB3	2	3	Scapula	1 (33.33)	1 (33.33)	1 (33.33)	1 (33.33)	—	—
S2/OB4	2	11	Femur (prox. epiph)	—	—	1 (9.09)	—	—	—
			Scapula	—	—	—	1 (9.09)	—	1 (9.09)
S2/OB5	1	2	Femur	1 (50)	1 (50)	—	—	—	—
S2/OB6	1	3	Tibia		1 (33.33)	—	1 (33.33)	—	—
S2/OB7	1	4	—	—	—	—	—	—	—

Furrowing was detected on 3.85% of the sample (n = 3). This alteration was recorded on the olecranon of an ulna, as well as on the glenoid cavity of a scapula and the proximal epiphyses of a femur. Crenulated edges were registered with the same percentages on the spine, medial border, and infraspinous fossa of scapulae (n = 2) and on the mid-shaft fragment of a tibia. Crushing was detected on one rib (1.28%) and longitudinal cracks were recorded on the spine of one scapula with the same rate.

**Figure 3:**
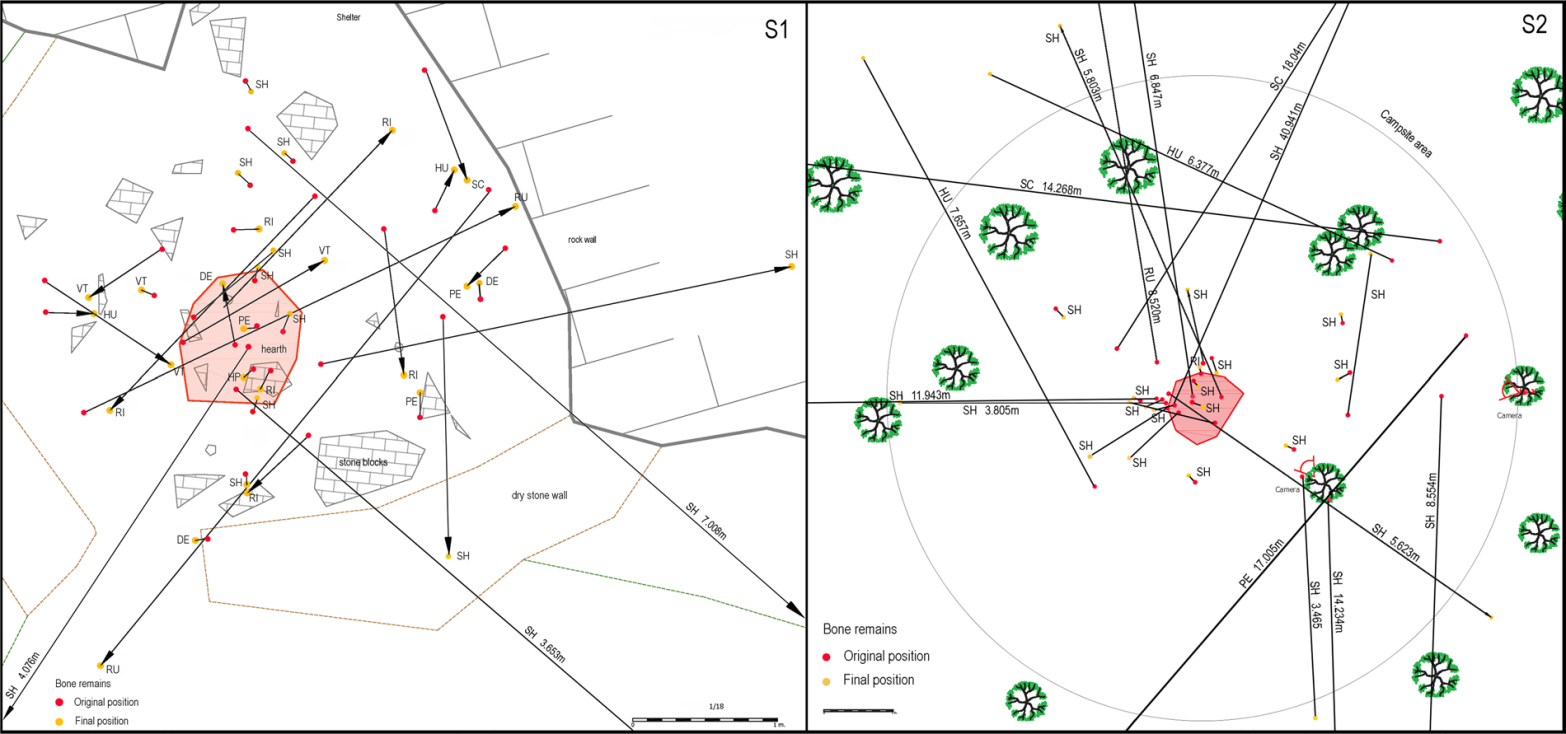
Superposition of all bone remain displacements from their original position to its final position in both sectors (S1/S2). Abbreviations: SH (mid-shaft fragment); PE (proximal epiphyses); DE (distal epiphyses); SC (scapula); RU (radius-ulna); HP (hemi-pelvis); RI (rib); VT (vertebra); HU (humerus). The red area corresponds to the hearth structure.

The general results are noticeable when assessing the specific area of the campsite. From a total of 78 skeletal elements recovered, 64 (82.1%) suffered translocations ranging from few centimetres to many metres. The furthest bone item was recovered at a distance of 40.94 metres (Fig. 3). Only half of the total recovered elements (n = 39; 50%) were located within the campsite area belonging to 6 out of 10 of the campsites (S1/OB1-OB2; S2/OB1-OB2-OB4 and OB7). The rest of the observations (n = 4) do not bear any bone remains inside the camp. Most of the remaining bones correlate with size class 4 (n = 29) and to a lesser extent with size class 2 (n = 12). The inner-site remains show a bias towards mid-shaft long bone fragments (n = 22), followed by distal and proximal epiphyses (n = 2 both) and hemi-pelvis (n = 1) regarding size class 4. As for size class 2, 6 ribs, 3 vertebrae, two humeri and one scapula were recorded. Furthermore, when individualizing the sites there are substantial differences. Mid-shaft fragments are the only survival skeletal elements at five out of six inner-campsite areas with the exception of S1/OB2 being the only one that bears small-sized bone remains. Bone damage was recorded in 3 bones (3.85% related to recovered bones) of size class 2 in the established area. One scapula, one vertebra, and one rib bear bone alteration in the form of pits was recorded.

It is worth noting that all damaged bones were recovered outside the hearth-related area. Only 3 lamb bones (1 dorsal vertebra, 1 rib and 1 scapula) from the influence area of the hearth showed isolated pits. It means that, after the carnivore ravaging, only 7.7% of the bones from the hearth-related area showed carnivore damage. However, all these bones come from the same observation (S1/OB2).

The cattle specimens from the hearth-related areas seem to correspond to those with higher bone-density. The tests revealed a strong correlation (r = 0.7036701, p-value = 0.0344), which could be indicating a carnivore preference for bone portions of large-sized animals with less density. The χ^2^ results (X-squared = 22.164, p-value = 0.008373) indicate that both categoric variables (bone portion and inside/outside the campsite) are not independent and, therefore, that the epiphyses have a high probability to disappear after the carnivore ravaging. The bone-density correlation applied to lamb data did not result in a significant value (r = 0.148003 p-value = 0.7796), which is the opposite of the cattle results. The χ^2^ results indicates a high likelihood of disappearance of lamb bones (X-squared = 36.461, p-value = 0.000002243).

**Figure 4:**
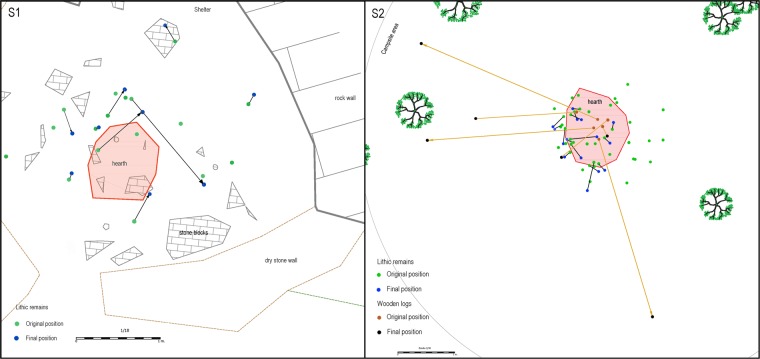
Superposition of lithic remains/burnt wooden logs and its displacements in both sectors (S1/S2). The red area corresponds to the hearth structure.

Scattering actions also affect lithic remains and some of the wooden logs from the hearth. With respect to the former, from the total of 60 stone tools placed around the hearth, a percentage of 41.6% (n = 25) belonging to 7 simulated campsites were shifted from their original emplacement. The range of displacements varies from 3.6 cm to 113.9 cm. Although the structured hearth was not disturbed, red foxes were able to interact with it removing some of the small logs used to make the fire. Six wooden logs were considerably displaced, ranging from 17.9 cm. to 338.5 cm (Fig. 4).

**Figure 5:**
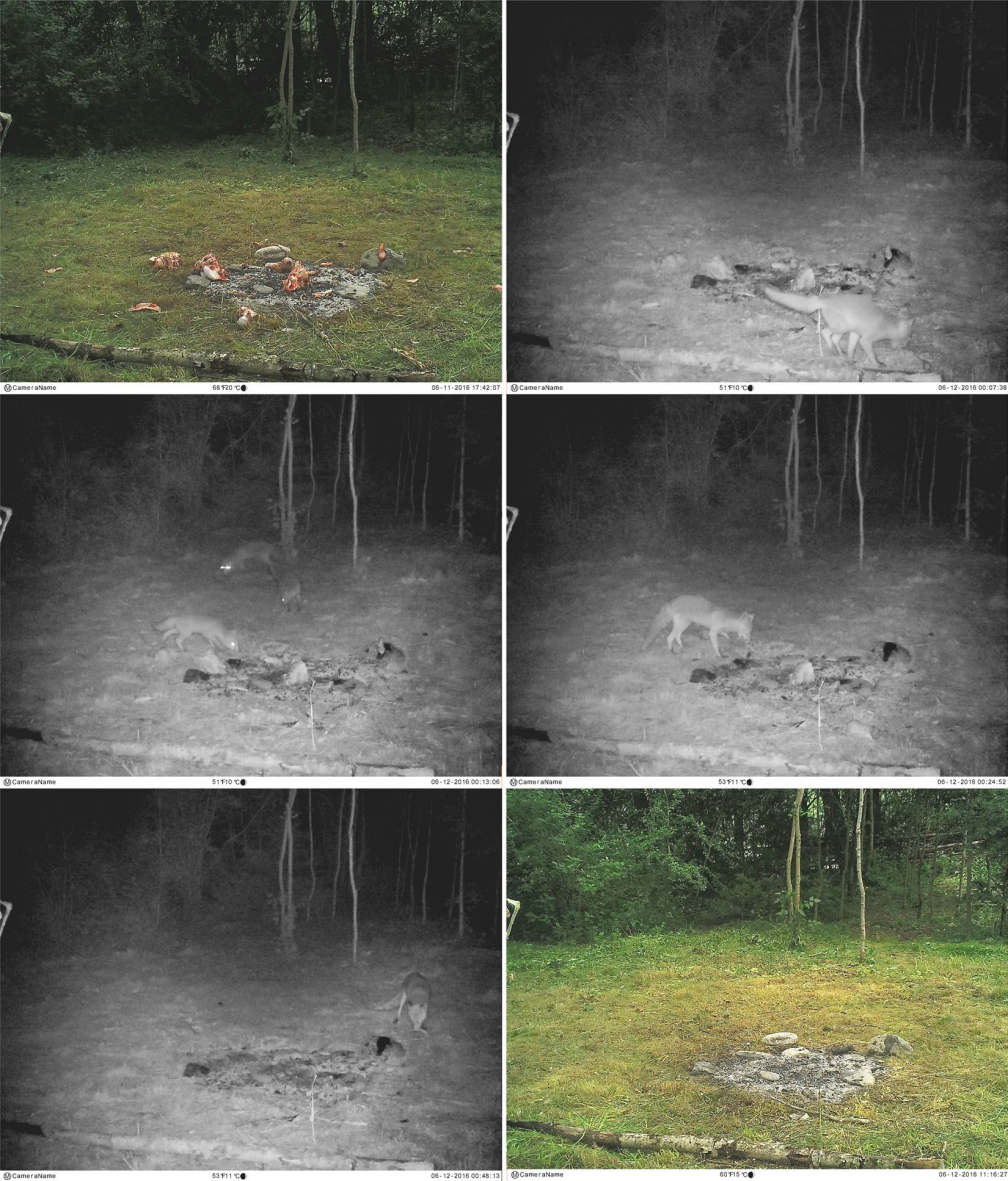
Sequence of pictures depicting the swiftness with which red foxes are able to dismantle an abandoned campsite. Notice the timing at the right bottom of the pictures (S2/OB1). The photo-trap was conducted using No Glow Infrared Trail cameras that are equipped with invisible black LED (Black Flash).

**Figure 6:**
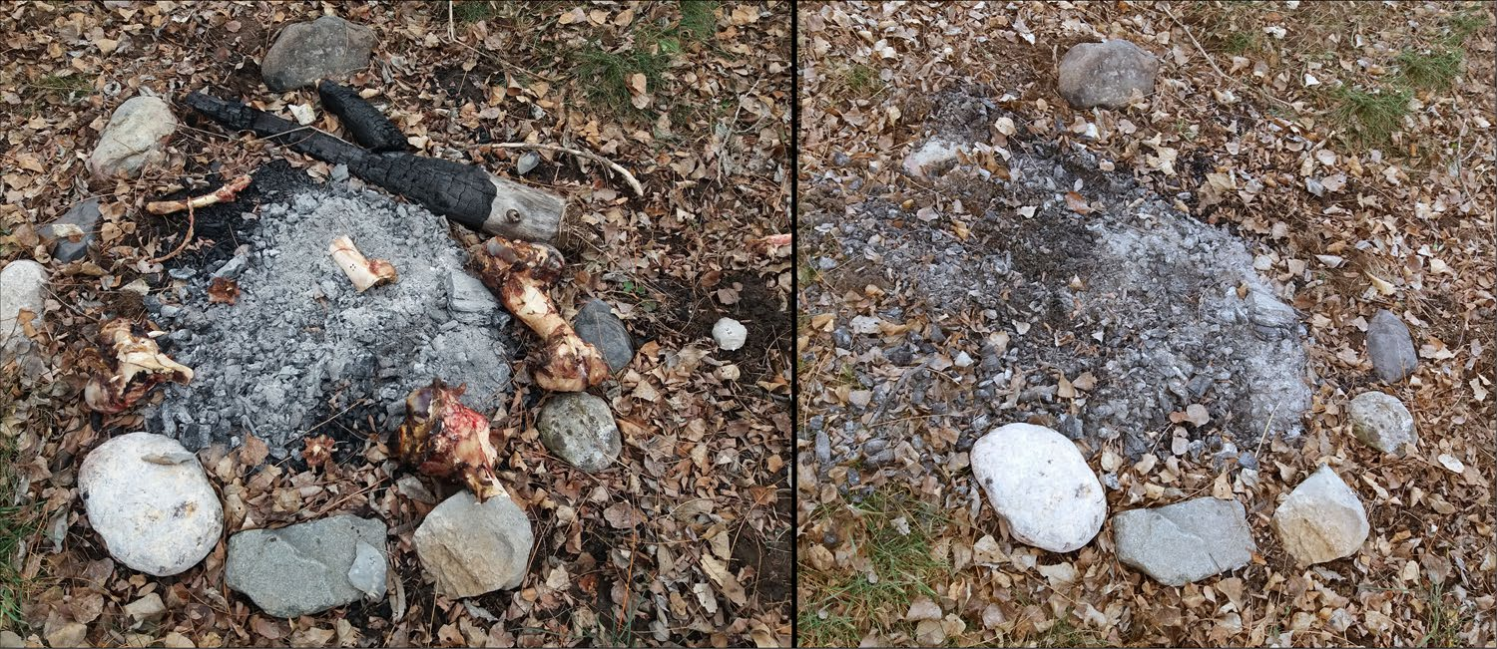
Image displaying the same picture of the central hearth three days after the simulated campsite was abandoned (S2/OB5).

The scavenging activities occurred during the first 5 days once the campsite was abandoned in all observations, with the exception of S1/OB2 (Table 6). In S2 looting actions occurred during the first 3 days, while in S1 there was a slight delay in OB1 and OB3 and a significant adjournment in OB2, as reported above. In this case, the first predator appeared after 28 days. Once discovered, small carnivores, primarily red fox, started removing as many skeletal elements as possible to secluded places, mainly epiphyses of large long bones, while most of the small bone remains were eaten at the place. The timing between those round trips took between 2 minutes to 23 minutes with an average of 7 minutes (Table 6). This variability is probably due to the distance covered. Wildlife cameras revealed different directions taken by red foxes that could indicate diverse random places to hide the remains. This predator behaviour caused the dismantling of the campsite in a few hours, mainly within the first nights after the abandonment of the site (Figs. 5 and 6). After these main predatory activities, some sporadic visits continued to occur, principally exploring but without any remarkable interference.

**Table 6:** Timing of the predatory activities showing campsite exposure, visits and round trips with the approximate average between round trips.

Sector/OB	Campsite setting up	Main carnivore	First visit	Time first visit	Round trips	Timing [min]	Last visit	Time last visit	Other secondary predators
S1/OB1	30/04/2016	Red fox	03/05/2016	6:19 PM	17	7	04/05/2016	1:27 AM	Pine marten
S1/OB2	28/05/2017	Red fox	25/06/2016	4:03 PM	10	7	13/07/2016	5:59 AM	Brown bear, pine marten
S1/OB3	05/05/2018	Red fox	10/05/2018	6:11 AM	11	8	21/05/2018	9:12 PM	Pine marten
S2/OB1	11/06/2016	Red fox^3^*	11/06/2016	11:12 PM	18	-	13/06/2016	4:25 AM	—
S2/OB2	22/10/2016	Red fox	22/10/2016	7:37 PM	17	7	25/10/2016	5:42 AM	—
S2/OB3	05/03/2017	Red fox	05/03/2017	8:40 PM	16	6	07/03/2017	6:31 AM	—
S2/OB4	17/09/2017	Red fox	18/09/2017	12:15 AM	22	5	23/09/2017	2:09 AM	Stone marten
S2/OB5	17/02/2018	Red fox	19/02/2018	8:20 PM	18	9	20/02/2018	10:37 AM	Common buzzard, griffon vulture, genet, stone marten
S2/OB6	28/07/2018	Red fox	29/07/2018	4:38 AM	18	6	30/07/2018	4:39 AM	Stone marten
S2/OB7	09/09/2018	Red fox	11/09/2018	4:42 AM	23	8	18/09/2018	5:00 AM	Stone marten, wild boar

OB = Observation period.

*In S2/OB1 three red foxes were recorded scavenging together, probably a female with two cubs, therefore it was nearly impossible to estimate the timing average due to the alternate round trips.

**Figure 7:**
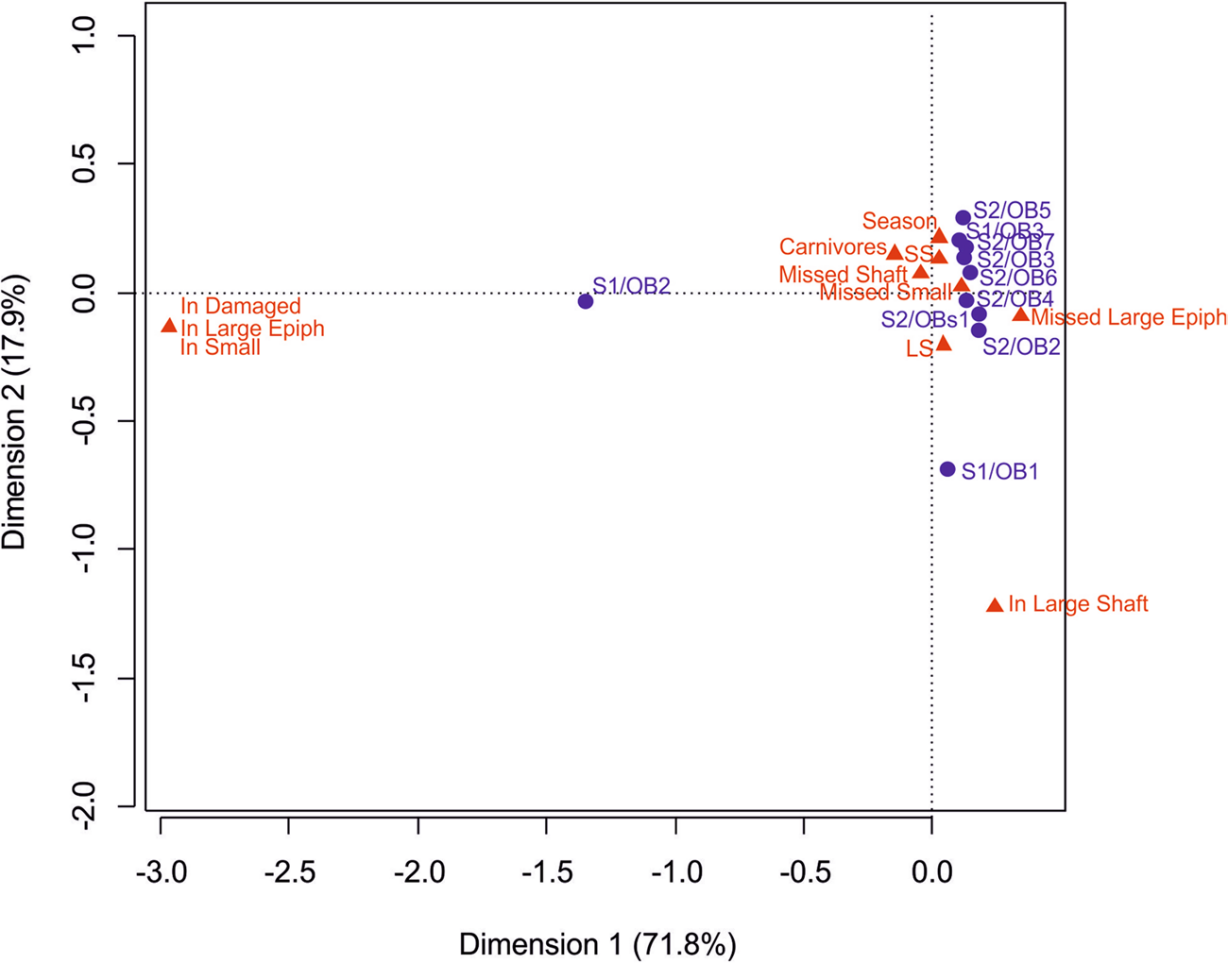
Component analysis (CA) of the OBs and variables (SS: Small size specimens; LS: Large size specimens; In: Inside the hearth-related area; Missed: Unrecovered specimens and specimens located outside the hearth-related area).

The contingency index indicates a high dependence of the analysed variables (χ^2^ = 259.13, p-value < 2.2e-16). CA indicates two groups of influence: the first is located at the right of Y-axis and very close to the origin; the second is further to the left of this axis and contains only three variables linked to elements from the hearth-related areas: epiphyses of large-sized animals, remains of small animals and damaged bones (Fig. 7). All the OBs are clustered in the first group, excepting the S1/OB2 that is located in the second group. It is important to note that this OB was the unique in which bears acted on the assemblage.

## Discussion

Neo-taphonomic studies were employed to assess the influence of scavenger distribution on hominin bone assemblages. We find hominin campsite refuse to be an attractive and easy meal for scavengers. Bone remains, including unbroken bones, ends and shaft fragments, frequently retain residual meat and grease. Thus, fossil hominin bone assemblage signatures are likely to have been overwritten by a range of scavengers and carnivores.

The most striking consequence after each actualistic series is the almost complete depletion of the epiphysis of long bones (*Bos taurus;* X-squared = 22.164, p-value = 0.008373) and the bones belonging to small-sized animals (*Ovis aries;* X-squared = 2.1696, p-value = 0.9035). Conversely, a large percentage of mid-shaft long bone fragments as a result of bone marrow extraction would remain on the simulated site. The absence of grease and nutrients makes these fragments less attractive to scavengers (e.g.,^10,17,19–21^). This fact leads to a high number of mid-shaft fragments (size class 4) at the campsite with hardly any carnivore alteration. In contrast, the small number of remote recovered bones (size class 2) bears significant carnivore damage. Thus, the carnivore activities at the site display a specific pattern regarding to both bone-absence/presence and bone-inflicted damage similar to those observed by Binford *et al*.^9^ and Blumenshine^10^ in their respective simulated sites. In these works, however, spotted hyena was the main disrupting agent, although mongooses, jackals and other scavengers were also involved. The disturbances documented by these authors were exclusively nocturnal and never confirmed through sightings, but the presence of hyenas was marked by their ‘wooping’ and ‘laughing’. The remaining skeletal elements showed an average of 15% of tooth-marks and a maximum incidence of 45%. Likewise, Bartram and Marean^35^ used ethnoarchaeological data from Kua foragers of the eastern Kalahari (Central District, Botswana) to deal with the Klasies River Mouth pattern. The bone assemblages collected and subsequently analysed among the Kua come from two different kinds of places: campsites and kill sites. The ravaging actions of hyenas focus on epiphyseal portions of large bovid while limb shaft fragments were relatively ignored in both sites. This phenomenon is common in most experiments and current observations, which have revealed that bone survival is density dependent in contexts without carnivore competition (e.g.,^13^ –in the case presented here, no competition between red foxes was observed). Thus, carnivores can significantly alter the skeletal profile of faunal assemblages generated by another primary agent, creating a potential equifinality problem for taphonomic analyses^12,36^.

The resulting bone assemblage pattern in our work is thoroughly altered by small-sized carnivores. As reported, one of the main interests of these small predators, specifically the red fox, is to move away many skeletal elements to secluded places^29,37–39^. The instinctive behaviour of these animals causes a remarkable degree of scattering, resulting in transport patterns that appear to be accentuated depending on variables like seasonality. Consistent with the previous studies, our remaining bone assemblage shows a 17.95% of bone inflicted damage and a 15.38% of recovered bones bearing tooth marks. Therefore, scavenging activities inflicted by small-sized carnivores can generate similar resulting scenarios as those perpetrated by large carnivores.

### Sector 1 (S1)

The red fox is the major taphonomic agent in Sector 1. The images display that once the campsite is discovered, red fox would start round trips to clear up the site. Some small skeletal elements would be engulfed at the site while the biggest anatomical portions (e.g., proximal and distal epiphyses of large-limb bones) would be carried away for later consumption (Supplementary Video S1). The remaining pattern comprises the total number of mid-shaft limb bone fragments together with the lithic remains. The only exception seems to be OB2, which was the least affected by predators. The date of the experiment could be one of the reasons of these carnivores’ behaviour. While OB1 and OB3 were set up and removed during spring season, OB2 was installed to the end-spring/early-summer season and removed in summer. This time lapse is concordant with the increasing availability of resources in the environment. As recorded in a previous study^38^ and in the current one, the lowest degree of bone alteration/scavenging activities by red fox has been registered in summer season. In S1/OB2, the images reveal the presence of different carnivore species typically ignoring the skeletal remains. Taphonomically speaking, this fact makes a low degree of alteration in both spatial disruption and bone modification. Between those carnivores (*Vulpes vulpes* and *Martes martes*), the presence of a brown bear (*Ursus arctos*) female with two cubs plays a significant role.

However, the results demonstrate opposing scenarios than the work conducted by Camarós *et al*.^22^ on the bears from *Parque de la Naturaleza de Cabárceno* (Penagos, Cantabria, Spain). In that study, the structured hearth and related assemblage was highly disturbed and virtually erased to the point of being no longer recognisable. The stones displaced around the hearth together with the stone tools were gnawed and scratched by several bears and the bones were carried out and not recovered. Conversely, our observations show one fleeting presence of a female with two cubs along the three-year study, specifically in OB2. The images display the female bear approaching cautiously toward the hearth without moving a single element while the cubs remain outside the shelter. They leave the place 1 min 34 seconds later leaving an untraceable presence (Supplementary Video S2). Moreover, we could register the presence of different brown bears nearby the campsite through the tracking systems. Six different wild-life cameras placed at different points and distances show 30 sightings of different individuals during the experiments’ timing (Table 7). Although their eyesight is relatively poor, bears can detect food sources from a huge distance (<10 km) because of their highly developed sense of smell^40,41^. Clean air in the Natural Parc of Alt Pirineu makes the scent of a campsite perceivable even for the human sense of smell at a relatively large distance as we could verify. Thus, their absence at the site can only be explained by their lack of interest or negative previous experiences. Those opposite scenarios might come from different methodologies. While our research develops under free-ranging wild animals, the former study deals with animals in a semi-free state, with around 70 individuals living in an enclosed area of 7.32 ha. The home range of a single individual in the Pyrenees varies from 8,300 to 92,300 ha depending on the genus, year, and the breeding period of females, while core areas vary from 1,990 to 66,000 ha^42^.

**Table 7:** Characteristics and positioning of six wild-life cameras showing bear sightings near the abandoned campsite in Sector 1.

Year	Season	Sector	OB	Campsite exposure	Camera location	Sighting date	Distance to campsite	Bear characteristics
2016	spring	1	1	30-april/28-may	Clavera	01-may	300 m	adult
					Marimanya	15-may	2 km	adult
					Port de Salau	17-may	3.1 km	adult
					Montgós	17-may	2 km	male and female+ 2 cubs (two years old)
					Bony mina	17-may	1 km	adult
					Montgós	23-may	2 km	female+2 cubs
					Raspamala	23-may	1 km	male
					Raspamala	24-may	1 km	indeterminate
					Port de Salau	26-may	3.1 km	adult
2017	end spring/summer	1	2	27-may/13-july	Port de Salau	27-may	3.1 km	adult male
					Bony mina	28-may	1 km	adult male
					Montgós	05-jun	2 km	adult male
					Clavera	06-jun	300 m	adult male
					Marimanya	29-jun	2 km	adult male*
					Raspamala	29-jun	1 km	adult male*
					Bony mina	30-jun	1 km	indeterminate
					Marimanya	30-jun	2 km	adult male*
					Raspamala	30-jun	1 km	adult male*
					Montgós	02-jul	2 km	adult*
					Port de Salau	05-jul	3.1 km	adult*
					Port de Salau	09-jul	3.1 km	female+2 cubs**
2018	spring	1	3	05-may/15-june	Clavera	06-may	300 m	adult male
					Montgós	22-may	2 km	adult male

OB = Observation period.

*29/06: same individual; 30/06: same individual; 02/07-05/07: probably the same individual.

**Probably the same female with two cubs recorded by our cameras at the campsite (S1/OB2).

### Sector 2 (S2)

As in Sector 1, red fox is also the major but not exclusive actor involved in Sector 2. Some different small-sized carnivores such as stone marten (*Martes foina*) and genet (*Genetta genetta*) also participated in the predatory actions (S2/OB 4–5) (Supplementary Video S3). In addition to those key taphonomic agents, a set of secondary small predators would participate in the feast. For instance, a small wood mouse (*Apodemus sylvaticus*) was recorded more than 200 metres away dragging a lamb vertebra that could not be retrieved (Supplementary Video S4) (S2/OB5). This bone element was previously moved away by a small carnivore and left near a small abandoned barn where it was taken by the wood mouse. A Eurasian buzzard (*Buteo buteo*) and a vulture (*Gyps fulvus*) feeding on small pieces of grease and meat were also recorded (S2/OB5) (Supplementary Video S4). Although its taphonomic footprints on the bones are hardly traceable, some skeletal elements suffered sensitive displacement under the pressure of the beak’s movement. Those bone remains were not recovered because of the subsequent looting actions of the small carnivores. A wild boar (*Sus scrofa*) was also recorded displacing the proximal epiphyses of a femur some centimetres while searching for maggots (S2/OB6) (Supplementary Video S4). Red fox is the only animal that interacted with the hearth. Once the attractive skeletal elements were transported and/or eaten, red fox would start scratching the ashes and charcoal displacing a few metres of some of the burnt small logs (S2/OB4–6) (Supplementary Video S5).

The bone assemblage pattern within the camp resulting from the looting actions of small-sized carnivores consists primarily of a collection of mid-shaft fragments belonging to caw limb bones together with the lithic remains. Six of the ten campsite areas bear only skeletal elements belonging to size class 4, and specifically mid-shafts. A large number of these items were barely displaced from its original position. However, there is a significant tendency to the disappearance of the limb bone epiphyses of size class 4 and all the bones of size class 2. Besides, no bone damage is observed in the hearth-related areas. This fact could represent a problem when interpreting the carnivore ravaging on the sites, since their presence could be undetectable. Nevertheless, a certain degree of diversity can be detected in the S1/OB2, which differs significantly from the other ones. The low presence of epiphyses and small-sized remains (some of them damaged) is a key characteristic to separate this OB in the CA. This hearth-related area was the unique that received the visit of brown bears, and this fact could have altered the behaviour of the usual scavengers, as observed by Faith *et al*.^13^ in carnivore competition contexts.

Considering our study as a simulated short-term occupation campsite, the resulting assemblage pattern presents a conspicuous similarity to some different archaeological sites identified as well as short-term or temporary occupational sites (e.g.,^23,43,44^). One of the bases associated with this type of lodging is the alternate presence of humans and carnivores and their corresponding traces on bone remains. Many of the archaeological sites described as short-term human occupations have also been interpreted as specialised hunting sites, thus supporting the idea of different subsistence strategies and territory management^23,44–47^. The continuous presence of large mammals in Middle Palaeolithic assemblages has pointed Neanderthals mainly as large game hunters^48,49^.

For example, the analyses of late Mousterian faunal remains from Gatzarria Cave (Ossas-Suhare, Pyrénées-Atlantiques, France^44^;) displays an overall pattern composed by a high number of shaft fragments against of the extremely poor representation of long bone epiphyses. This fact is attributed to some destruction of low-density bone portions at the site. The absence of small-size animals in the assemblage leads one to conclude that these prey species were not an important element of human diet at the site. The low percentage of carnivore modified remains is also comparable, with 2.6% of ungulate diaphysis exhibiting carnivore damage versus 6.4% in our work. Moreover, according to body size rule^50,51^, Neanderthals at Garratzia indicated a preference for some resources and exploited mainly large mammals, specifically red deer. The similarities between those final assemblages result in different explanations exposing a complex subject matter of interpretation. The analyses of level J (sublevels Ja and Jb) from Abric Romaní (Capellades, Barcelona, Catalonia)^23^ reveal similar archaeological features, within which medium- and large-sized ungulates (red deer and horse) are the dominant taxa. The high degree of limb bone breakage, the low percentage of axial skeletal elements and practically a total lack of epiphyses, has been interpreted in this site as the result of a human primary access to the ungulates, the preferential transport of limbs and cranial elements and an intensive pattern of consumption related to the regular use of fire. As in the previous work, the assemblage also presents a low degree of bone damage inflicted by carnivores. The frequency and patterning of carnivore damage suggest that many of the animal marks on ungulate specimens may represent secondary access to the remains. However, while the type of cut marks on the diaphysis implies that limb bones were brought complete to the site the absence of limb bone epiphyses cannot be explained in the same way. Although anthropogenic processing and consumption patterns seem to be the most plausible explanation according to the researchers, they do not rule out the possibility of carnivore activities after human occupations.

## Conclusions

Resource availability in the environment influences the survival capabilities of species and can condition migratory movements. It is evident that European ecosystems of the past are not the same as current ones, since there are many carnivore and large-bodied ungulate species that have disappeared (e.g.,^52^). Thus, it is not easy to make realistic approximations of aspects related to carnivore behaviour in relation to the prey versus predator density in the past. Nevertheless, there are works that propose species distribution models through mathematical approaches that could help assess ecological relationships between different biological entities in studied territories (e.g.,^53,54^). This work aims to be a starting point for evaluating a taphonomic problem that we consider recurrent in most archaeological sites and that conditions many interpretations made so far. This study calls attention to the significant role played by small-sized carnivores on the taphonomic signature of abandoned hominin campsites. The results show that the effects of small-sized scavenger disturbances are noteworthy, have direct implications on zooarchaeological analyses and, therefore, on the final interpretation of archaeological bone assemblages. Understanding bone modification processes in experimental contexts becomes essential to recognise analogous processes in the archaeological record, considering the relevance of our hominin behavioural assumptions from faunal remains.

Although anthropogenic structures, such as the hearth, are seldom disturbed, small-sized carnivores (mainly red foxes) are capable of modifying bone assemblages to a high degree while leaving extremely few traces. Resulting bone patterns are primarily characterized by mid-shaft long bone fragments of size class 4 with a very low percentage of carnivore-induced modifications. Regarding spatial distribution, a high percentage of bone remains suffered displacement ranging from a few centimetres to several metres. Lithic remains, together with some small wooden logs, were also relocated to a lesser extent.

The usual exploitation of medium- and large-sized ungulates on short-term human occupations might be one plausible explanation for the pattern left in this study. However, our results do not support this argument. The experimental data presented provides significant understanding of the complexity faced when dealing with bone assemblages discarded by hominins. Considering that the behaviour of small carnivores has consistent scavenging/scattering patterns, the modelling of these patterns can help determine the formation of palimpsests in archaeological sites.

## Supplementary information

Supplementary Video S1.

Supplementary Video S2.

Supplementary Video S3.

Supplementary Video S4.

Supplementary Video S5.
